# Evaluating the role of RAD52 and its interactors as novel potential molecular targets for hepatocellular carcinoma

**DOI:** 10.1186/s12935-019-0996-6

**Published:** 2019-11-06

**Authors:** Ping Li, YanZhen Xu, Qinle Zhang, Yu Li, Wenxian Jia, Xiao Wang, Zhibin Xie, Jiayi Liu, Dong Zhao, Mengnan Shao, Suixia Chen, Nanfang Mo, Zhiwen Jiang, Liuyan Li, Run Liu, Wanying Huang, Li Chang, Siyu Chen, Hongtao Li, Wenpu Zuo, Jiaquan Li, Ruoheng Zhang, Xiaoli Yang

**Affiliations:** 1 Center of Diabetic Systems Medicine, Guangxi Key Laboratory of Excellence, Guilin Medical University, Guilin, Guangxi China; 2 Scientific Research Center, Guilin Medical University, Guilin, Guangxi China; 3 Medical Scientific Research Center, Guangxi Medical University, Nanning, Guangxi China; 4 College & Hospital of Stomatology Guangxi Medical University, Nanning, Guangxi China; 5 Department of Pathology, The First Affiliated Hospital of Guangxi Medical University, Nanning, Guangxi China; 6 College of Pharmacy, Guangxi Medical University, Nanning, Guangxi China; 7 Genetic and Metabolic Central Laboratory, The Maternal and Children Health Hospital of Guangxi, Guangxi, China; 8 Department of Pathophysiology, Guangxi Medical University, Nanning, Guangxi China; 9 Medical Science Laboratory at Liuzhou Worker’s Hospital, Liuzhou, Guangxi China; 10 Penn State College of Medicine, Hershey, PA USA

**Keywords:** Hepatocellular carcinoma, RAD52, DNA repair, Diagnostic marker, Molecular interaction

## Abstract

**Background:**

Radiation sensitive 52 (RAD52) is an important protein that mediates DNA repair in tumors. However, little is known about the impact of RAD52 on hepatocellular carcinoma (HCC). We investigated the expression of RAD52 and its values in HCC. Some proteins that might be coordinated with RAD52 in HCC were also analyzed.

**Methods:**

Global RAD52 mRNA levels in HCC were assessed using The Cancer Genome Atlas (TCGA) database. RAD52 expression was analyzed in 70 HCC tissues and adjacent tissues by quantitative real-time PCR (qRT-PCR), Western blotting and immunohistochemistry. The effect of over-expressed RAD52 in Huh7 HCC cells was investigated. The String database was then used to perform enrichment and functional analysis of RAD52 and its interactome. Cytoscape software was used to create a protein–protein interaction network. Molecular interaction studies with RAD52 and its interactome were performed using the molecular docking tools in Hex8.0.0. Finally, these DNA repair proteins, which interact with RAD52, were also analyzed using the TCGA dataset and were detected by qRT-PCR. Based on the TCGA database, algorithms combining ROC between RAD52 and RAD52 interactors were used to diagnose HCC by binary logistic regression.

**Results:**

In TCGA, upregulated RAD52 related to gender was obtained in HCC. The area under the receiver operating characteristic curve (AUC) of RAD52 was 0.704. The results of overall survival (OS) and recurrence-free survival (RFS) indicated no difference in the prognosis between patients with high and low RAD52 gene expression. We validated that RAD52 expression was increased at the mRNA and protein levels in Chinese HCC tissues compared with adjacent tissues. Higher RAD52 was associated with older age, without correlation with other clinicopathological factors. In vitro, over-expressed RAD52 significantly promoted the proliferation and migration of Huh7 cells. Furthermore, RAD52 interactors (radiation sensitive 51, RAD51; X-ray repair cross complementing 6, XRCC6; Cofilin, CFL1) were also increased in HCC and participated in some biological processes with RAD52. Protein structure analysis showed that RAD52–RAD51 had the firmest binding structure with the lowest E-total energy (− 1120.5 kcal/mol) among the RAD52–RAD51, RAD52–CFL1, and RAD52–XRCC6 complexes. An algorithm combining ROC between RAD52 and its interactome indicated a greater specificity and sensitivity for HCC screening.

**Conclusions:**

Overall, our study suggested that RAD52 plays a vital role in HCC pathogenesis and serves as a potential molecular target for HCC diagnosis and treatment. This study’s findings regarding the multigene prediction and diagnosis of HCC are valuable.

## Background

Radiation sensitive 52 (RAD52) is a DNA-binding protein that mediates the repair of DNA double-strand breaks (DSBs). DSBs are among the most severe types of DNA damage in eukaryotes and lead to apoptosis, necrocytosis, and tumor formation if not repaired promptly and accurately [1]. There are two main DSB repair pathways: homologous recombination (HR) and nonhomologous end-joining (NHEJ). RAD52, an important HR protein, belongs to the RAD52 epistasis group, which includes RAD50, RAD51, RAD52, RAD54, RAD55, RAD57, RAD59, MRE11, and XRS2 [2]. Among all of the members of this group, RAD52 has the strongest effect on HR and DNA repair in *Saccharomyces cerevisiae* [3]. Apart from defects in DSB repair, RAD52 mutants also show a deficiency in mating-type switching, meiosis, spore viability, and homologous DNA integration into the genome [4].

Hepatocellular carcinoma (HCC) is the sixth most common cancer worldwide and is the third leading cause of cancer mortality worldwide [5, 6]. Risk factors associated with HCC include cirrhosis, chronic hepatitis B or C infection, alcohol, diabetes, obesity, aflatoxin B1, and some inherited metabolic disorders [6–10]. At present, serum alpha-fetoprotein (AFP) detection, CT scan, and B-mode ultrasound are common tools for the early diagnosis of HCC; however, the misdiagnosis rate is high [10]. The major therapy for HCC is surgical resection. Liver transplantation, radiotherapy, ablative therapies and other therapies are also applied [11, 12]. However, a high incidence of tumor recurrence and intrahepatic metastasis is clinically common after surgical resection [12]. Due to the difficulty of early diagnosis and effective treatment, the 5-year survival rate of HCC is only approximately 7%. Up to 600,000 people die of HCC every year in China [5]. Therefore, investigating the pathogenesis of HCC is of great significance for reducing the incidence of HCC and increasing the cure rate for this disease.

Defects in HR lead to genomic instability and are associated with cancer predisposition [13, 14]. A key step in HR is the formation of RAD51 nucleoprotein filaments. RAD52 was found to interact with RAD51, which suggested its role in RAD51-related DNA recombination and repair [15–17]. Some studies revealed RAD52 to be part of an independent and alternative repair pathway of DBSs and DNA replication stalling independent of BRCA2 [17–19]. Furthermore, it has been proven that RAD52 is involved in the response to oncogene-induced DNA replication stress [20]. New evidence suggests that RAD52 is essential for maintaining tumor genome integrity [2]. Several SNPs in RAD52 may be linked to the risk of multiple cancers, including breast cancer, lung cancer, thyroid cancer, head and neck cancers, and ovarian cancer [21–25]. High expression of RAD52 was detected in tumor cells, particularly in the lung squamous cell carcinomas and nasopharyngeal carcinoma tissues in previous studies [7, 26]. The RAD52 functional SNP rs7963551 was found to contribute to the susceptibility to HCC [27]. However, little or nothing is known about the role of RAD52 in HCC pathogenesis.

Our previous study showed that RAD52 and its interactome, consisting of several NHEJ proteins, were involved in HCC pathogenesis [28]. In this study, the impact of RAD52, an important HR protein, was assessed in HCC. We used RNA-seq data from The Cancer Genome Atlas (TCGA) database to analyze the transcriptional expression of RAD52 in HCC. Then, the mRNA and protein expression levels were detected in the paired HCC and adjacent tissues. The correlations between RAD52 expression and risk factors, as well as the clinicopathological characteristics of HCC, were analyzed. And the function of RAD52 in HCC cell lines was also investigated. Simultaneously, the String database was used to perform enrichment and functional analysis of RAD52 and its interactome. Cytoscape and Hex8.0.0 help us analyze the protein–protein interaction of RAD52 and its interactome. Moreover, we also analyzed the expression of these proteins, which may cooperate with RAD52 in HCC development and discussed their clinical roles.

## Materials and methods

### Data collection and analysis from the TCGA liver hepatocellular carcinoma dataset

Data collection and analysis were performed as described previously [6]. Briefly, clinical information and RNA sequencing (RNA-seq) data of HCC patients from the TCGA liver hepatocellular carcinoma (LIHC) dataset were downloaded from the Xena Public Data Hubs (https://xenabrowser.net/). Level 3 data were used. RNA-seq data were quantified using RNA-seq by expectation-maximization (RSEM).

### Patients and specimens

Collection and analysis of human clinical specimens occurred at the First Affiliated Hospital of Guangxi Medical University. 70 patients with HCC were enrolled. And there were 70 HCC tissues and 70 paired adjacent (peritumoral) tissues collected from the patients. All patients had complete medical history data. The criteria for inclusion in the study were as follows: (1) no anticancer treatment or distant metastasis prior to surgery; (2) no concurrent autoimmune diseases, HIV, or syphilis; and (3) available clinicopathological information. Histopathological diagnosis was made on routinely processed H&E sections. Demographics and clinicopathological factors for all specimens used in this study are summarized in Table 1. All the research project has been approved by the Ethics and Human Subjects Committee (EHSC) of Guangxi Medical University and conformed to the provisions of the Declaration of Helsinki. Informed consent was obtained from each patient included in the study.

**Table 1:** Correlation between the factors and clinicopathological characteristics in the Chinese population (n = 70)

Clinical features	Case	RAD52 positive rate (%; mean ± SD)	P-value
Sample			
HCC	70	65.600 ± 25.079	P < 0.05*
Adjacent tissue	70	48.057 ± 26.928	
Age (years)			
≤ 45	28	57.036 ± 28.299	P < 0.05*
> 45	42	71.310 ± 21.157	
Gender			
Male	61	66.918 ± 24.841	P > 0.05
Female	9	56.667 ± 26.339	
Smoking			
Yes	35	63.486 ± 26.579	P > 0.05
No	35	67.714 ± 23.681	
Drinking			
Yes	38	65.316 ± 26.366	P > 0.05
No	32	65.938 ± 23.876	
HBsAg infection			
Yes	50	68.700 ± 25.948	P > 0.05
No	20	57.850 ± 21.431	
AFP (ng/ml)			
≤ 20	20	66.100 ± 21.885	P > 0.05
> 20	50	65.400 ± 26.454	
Tumor size (cm)			
≤ 5	12	53.750 ± 23.848	P > 0.05
> 5	6	45.000 ± 32.711	
Unknown	52	70.712 ± 22.667	
Clinical stage			
I + II	32	63.656 ± 25.299	P > 0.05
III + IV	38	67.237 ± 25.113	
ALT			
≤ 40	38	64.083 ± 27.055	P > 0.05
> 40	32	70.517 ± 22.494	
AST			
≤ 40	28	65.280 ± 25.370	P > 0.05
> 40	42	68.000 ± 25.263	

*RAD52* radiation sensitive 52, *HCC* hepatocellular carcinoma, *AFP* alpha fetoprotein, *HBsAg* hepatitis B surface antigen, *ALT* glutamic-pyruvic transaminase, *AST* aspartate transaminase, *SD* standard deviation

* P < 0.05

### Reverse transcription polymerase chain reaction

Total RNA from HCC samples was extracted with TRIzol kit (Invitrogen, CA). The RNA was converted to cDNA using the PrimeScript RT reagent Kit with gDNA Eraser (Takara, JPN). The quantitative real-time PCR (qRT-PCR) protocol was performed on the StepOnePlus^®^ (Applied Biosystems, CA) as follows: denaturation at 95 °C for 10 min followed by 40 cycles of 95 °C for 15 s and 52–56 °C for 60 s. The concentration of mRNA was determined by fluorescence detection with SYBR Green Master Mix (Roche, GER) in triplicate and normalized to the expression of the housekeeping gene, β-actin. The primers used for qRT-PCR amplification were

5′-CAATTCTTGGAGGACGTGAC-3′(forward) and

5′-TGACCCTCAATGTAGCACAC-3′(reverse) for RAD52;

5′-CTCCATCCTGGCCTCGCTGT-3′(forward) and

5′-GCTGTCACCTTCACCGTTCC-3′ (reverse) for β-actin;

5′-CACCGCCCTTTACAGAACA-3′(forward) and

5′-GGGATCAGCAGCAAACATC-3′(reverse) for RAD51;

5′-GCGAGCACTCAGCAGGTTA-3′(forward) and

5′-GGTTCATTGTTTCCCGATA-3′(reverse) for X-ray repair cross complementing 6 (XRCC6);

5′-GCCGCTATGCCCTCTATGAT-3′(forward) and

5′-CTTGACCTCCTCGTAGCAGT-3′(reverse) for cofilin (CFL1).

### Western blotting

Tissues were lysed with radioimmunoprecipitation assay (RIPA) buffer, and denatured samples were prepared for immunoblotting. Target protein was separated by 12% SDS-PAGE and then transferred onto a PVDF membrane. Detection of the protein was performed using mouse anti-human RAD52 antibody (1:500 dilution) prior to incubation of the membrane with the appropriate secondary antibody. A FluorChem HD2 chemiluminescence system (Proteinsimple, CA) was used for protein visualization.

### Immunohistochemistry

Tissues were paraffin-embedded and sectioned. Paraffin-embedded sections (5 μm) were deparaffinized in three xylene washes followed by a graded alcohol series, antigen retrieval was performed with 10 mM sodium citrate buffer, and sections were blocked with blocking solution for 15 min at RT. Sections were incubated with primary antibody against RAD52 (1:800) overnight at 4 °C, followed by incubation with biotinylated secondary antibodies for 1 h at RT after washing in phosphate-buffered saline. Sections were developed with DAB reagent, counterstained with hematoxylin, dehydrated with ethanol and xylene, and mounted in resin blocks. Then, five high-power fields were randomly chosen in each section for assessment of RAD52, and at least 300 cells were counted per field. The positive rate was calculated based on the intensity of immune staining and the quantity of stained cells. The positive rate was evaluated by two independent pathologists.

### Cell lines and cultures

The human HCC cell lines SMCC-7721 (7721), MHCC 97H (97H), MHCC 97L (97L), Huh7, HepG2 (G2), HCC-LM3 (LM3), and BEL7404 (7404) were purchased from the Cell Bank of the Chinese Academy of Sciences (Shanghai, China). The cells were grown in high-glucose Dulbecco’s Modified Eagle Medium (DMEM; Gibco Company, USA) supplemented with 10% fetal bovine serum (FBS; Gibco Company, USA) and 1% penicillin/streptomycin. All the cultures were incubated at 37  °C with 5% CO_2_.

### Cell transfection

RAD52 over-expression plasmid was transferred into 293T cells using Lipofectamine 3000 (Invitrogen, Thermo Fisher Scientific, Inc.) and the virus packaging plasmid was preserved at 37 °C for 48 h. Then the lentivirus were packaged in 293T cells and collected from the supernatant. Huh7 cells were seeded on 6-well plates and incubated at 37 °C for a further 24 h prior to transfection. After concentrated and titrated, the virus solution was used to infect Huh7 cells. Following 48 h of transfection and screened by puromycin, the Huh7 cells were used for subsequent experiments.

### Cell growth assay

The proliferation ability of cells was detected by CCK-8 test. The cells were divided into RAD52 over-expression group and blank group. Their densities were adjusted to 1.5 × 10^3^ cells/ml and inoculated in 96 well plates with 100 μl/well. Three parallel holes were set in each group and incubated at 37 °C with 5% CO_2_ for 24 h, 48 h, 72 h, 96 h, 120 h. Then 10 μl CCK-8 was added into each well and cultured for 2 h. The optical density (OD) value was measured at 450 nm at the same time, and the proliferating ability of the cells was analyzed using the measured OD value.

### Scratch wound assay

Huh7 cells, cultured in serum-free DMEM complete media, were seeded in 6-well plates (7–8 × 10^6^ cells/well) for 24 h, during which time the cells adhered to the well and reached 80% density. Gently and slowly scratch the monolayer with a new 10 μl pipette tip across the center of the well. Scratch another straight line perpendicular to the first line to create a cross in each well. After 0, 24, 48, 72, 96 h, incubation at 37 °C, we took photos on a microscope. Images of the wound region in 10 random fields were captured using a light microscope (Olympus, Japan) at × 100 magnification. Each experiment was repeated at least three times.

### Functional and pathway enrichment analyses

Gene ontology (GO) analyses and Kyoto Encyclopedia of Genes and Genomes (KEGG) pathway enrichment analyses were conducted on genes using the Search Tool for the Retrieval of Interacting Genes (STRING, https://string-db.org/). Protein–protein interaction (PPI) network and functional annotation of genes were generated. Modules in PPI were then screened using molecular complex detection (MCODE) in the Cytoscape software package. *P ≤ 0.05 was considered to be statistically significant.

### Molecular docking

The 3D structure of ligand-proteins (RAD51, XRCC6, CFL1) and receptor-protein (RAD52) were found in the Protein Data Bank (PDB) database. Based on the PDB files, computational docking between RAD52 and its interactome was performed using HEX 8.0.0, a program that can dock interactive proteins. The Correlation Type was set as Shape + Electro, and the Final Search was set as 30. The program utilizes FFT correlation with the Gaussian density representation of the protein shape and spherical polar coordinates [29] (Additional file 1).

### Statistical analysis

All statistical calculations were performed with SPSS version 22.0 (IBM). Data were plotted as the mean ± standard deviation (x ± SD). Welch’s t-tests were used to compare two groups of data. Analysis of variance (ANOVA) and Pearson’s Chi-square test were used for correlation analyses. The receiver operating characteristic curve (ROC) and the area under the curve (AUC) were used to estimate the diagnostic value of different genes for HCC. Pairwise comparison of ROC curves was conducted by MedCalc. v9.2.0.1. The Kaplan–Meier survival analysis was used to estimate the overall survival (OS) and recurrence-free survival (RFS) curves. According to the median expression level of RSEM of different genes, patients were divided into two groups (high and low) for survival analysis. ImageJ was used for the semiquantification of the results of Western blotting. *P ≤ 0.05 indicated statistical significance in all analyses.

## Results

### RAD52 analysis based on the TCGA database

**Fig. 1:**
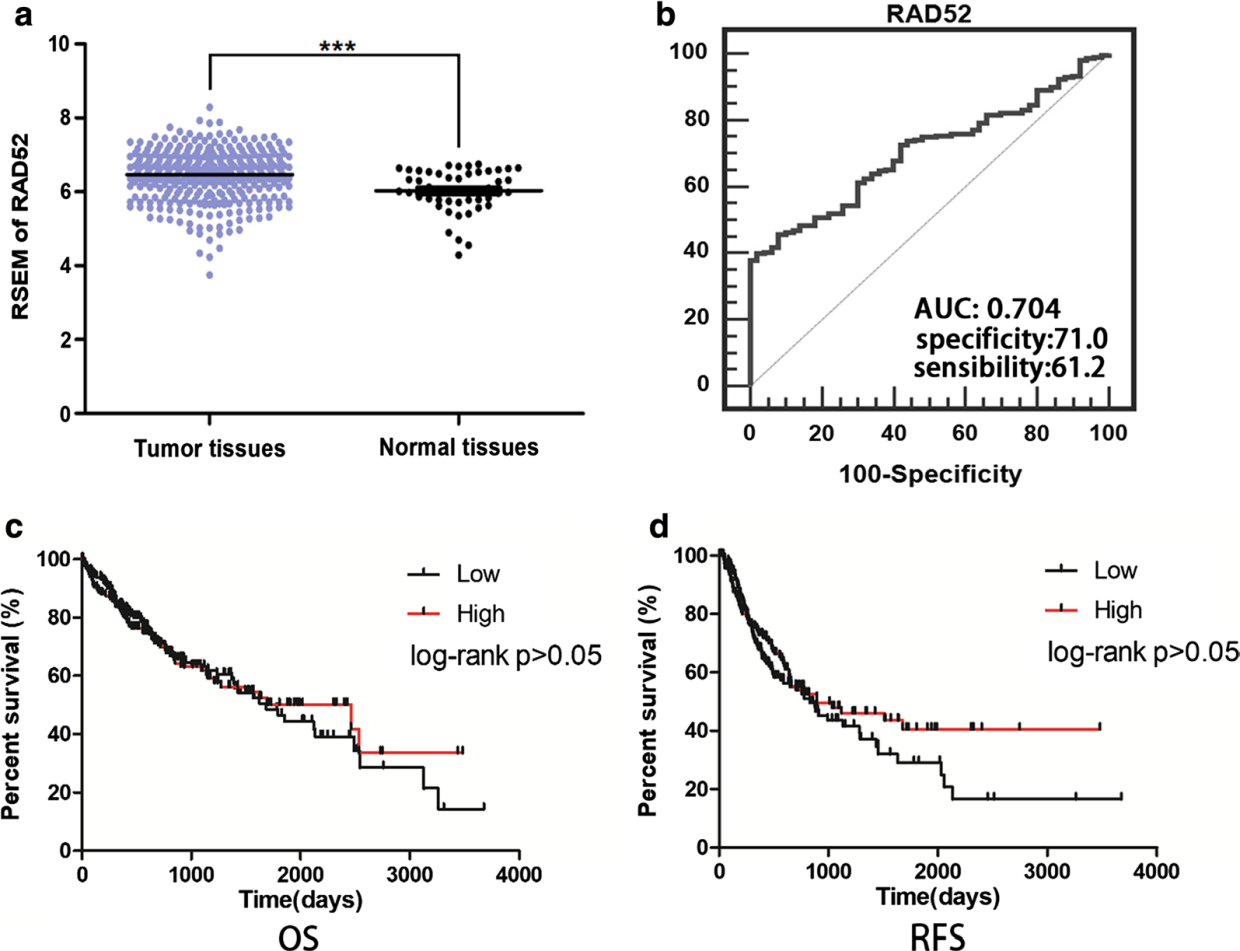
Data mining of RAD52 in the TCGA database. **a** RAD52 mRNA expression in HCC tissues and normal tissues. RNA-seq data were quantified using RSEM. Significant differences were observed between HCC tissues and normal tissues. Higher expression of RAD52 in HCC tissues than in normal tissues was obtained (***P ≤ 0.001). **b** Receiver-operation characteristic (ROC) curve of RAD52. The AUC of RAD52 was 0.704. The sensitivity and specificity were 61.20% and 71.00%, respectively. **c** Overall survival (OS) curve according to RAD52 expression in HCC. **d** Recurrence-free survival (RFS) of RAD52 expression in HCC

RAD52 expression in 413 tissue samples (50 normal tissues and 363 tumor tissues) at the mRNA level was obtained from the TCGA database in this research. RAD52 expression levels in HCC were higher than in normal tissue (Fig. 1a). An ROC curve was generated by plotting sensitivity versus specificity (Fig. 1b). The AUC value of RAD52 was 0.704 (*P ≤ 0.05), with an optimal cut-off point of 6.38. The sensitivity and specificity were 61.20% and 71.00%, respectively [30].

Moreover, we analyzed the relationship between the RAD52 level in HCC and various clinical, epidemiological, and pathological variables. The results showed that males were more susceptible than females. Except for gender, none of the clinical HCC features, such as AFP, hepatitis B surface antigen (HBsAg) infection, clinical stage, Child–Pugh classification, lymph node and metastasis, were significantly correlated with the RAD52 level (Table 2).

**Table 2:** Correlation between the factors and clinicopathologic characteristics in HCC in TCGA dataset (n = 363)

Clinical features	Case	RAD52 level (RSEM; mean ± SD)	P value
Sample			
LIHC	363	6.4606 ± 0.6778	P < 0.05*
Normal	50	6.0250 ± 0.5742	
Age at diagnosis (years)			
> 45	314	6.4371 ± 0.6918	P > 0.05
≤ 45	48	6.6329 ± 0.5475	
Unknown	1		
Gender			
Male	246	6.3721 ± 0.6948	P < 0.05*
Female	117	6.6466 ± 0.6020	
The AFP in serum			
> 20 ng/ml	129	6.5403 ± 0.6778	P > 0.05
≤ 20 ng/ml	143	6.4050 ± 0.6319	
Unknown	91		
Clinical stage			
I–II	251	6.4353 ± 0.6493	P > 0.05
III–IV	88	6.5033 ± 0.7782	
Unknown	24		
Child–Pugh classification			
A	213	6.4195 ± 0.7080	P > 0.05
B	21	6.4685 ± 0.5537	
C	1		
Unknown	128		
HBsAg infection			
Yes	226	6.4981 ± 0.6733	P > 0.05
No	122	6.4139 ± 0.6543	
Unknown	15		
Lymph node			
N0	246	6.4788 ± 0.6713	P > 0.05
N1	3	6.8001 ± 1.0916	
Unknown	114		
Metastasis			
M0	260	6.4512 ± 0.6827	P > 0.05
M1	4	6.0975 ± 0.5427	
Unknown	99		

*TCGA* The Cancer Genome Atlas, *LIHC* liver hepatocellular carcinoma, *HCC* hepatocellular carcinoma, *RSEM* RNA-seq by expectation-maximization, *RAD52* radiation sensitive 52, *AFP* alpha fetoprotein, *HBsAg* hepatitis B surface antigen, *SD* standard deviation

* P < 0.05

The OS and RFS results indicated that there was no difference in the prognosis between high and low RAD52-expressing patients (P > 0.05) (Fig. 1c, d).

### RAD52 expression in HCC tissues and adjacent tissues

**Fig. 2:**
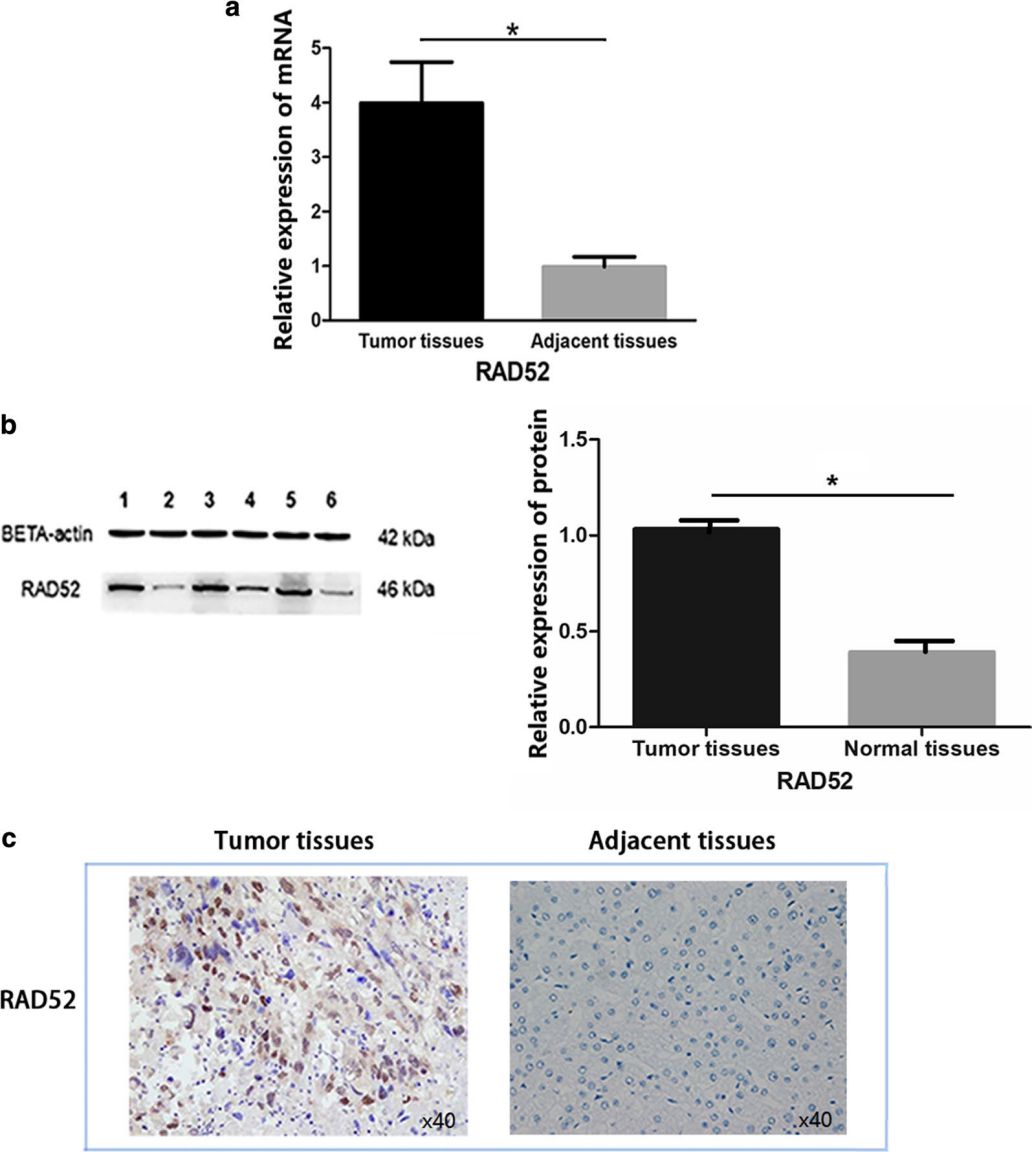
RAD52 expression was significantly upregulated in Chinese HCC tissues compared to their paired adjacent (peritumoral) tissues. **a** mRNA expression of RAD52 detected by qRT-PCR in HCC. Upregulated mRNA was shown in HCC tissues (*P ≤ 0.05). **b** RAD52 protein levels were measured by Western blotting. Lanes 1, 3, and 5 represent cancer tissues. Lanes 2, 4, and 6 represent adjacent tissues. Increased RAD52 protein expression was observed in HCC tissues. **c** RAD52 immunohistochemical testing in 70 paired HCC tissues and adjacent nontumor tissues. Representative IHC images of RAD52 expression are shown in the panel. The left is HCC tissues with high expression. The right is nontumor tissues with low expression

Further enrolled cohort study was performed in Chinese HCC patients, which provided HCC and paired adjacent (peritumoral) tissues. The mRNA expression level of HCC and paired adjacent tissues was compared by qRT-PCR and normalized to beta-actin. The results showed that RAD52 mRNA was increased in HCC tissues (*P ≤ 0.05, Fig. 2a). Western blotting results also indicated that RAD52 was higher in HCC tissues than that in adjacent tissues at the protein level (*P ≤ 0.05, Fig. 2b). The immunohistochemistry results indicated positive immunostaining for RAD52 in all HCC samples, with an average positive rate of 65.600 ± 25.079%. In adjacent samples, the average positive rate was 48.057 ± 26.928% (Table 1, Fig. 2c).

### Association between RAD52 expression and clinicopathological features of HCC patients

As shown in Table 1, the expression of RAD52 protein in HCC patients was related to age (*P ≤ 0.05). However, no association was observed between RAD52 expression and gender, smoking, drinking, tumor size, serum AFP level, HBV infection, clinical stage, glutamic-pyruvic transaminase (ALT), or Aspartate transaminase (AST) (P > 0.05).

### Over-expressed RAD52 promotes cell proliferation, migration in cultured Huh7 cells

**Fig. 3:**
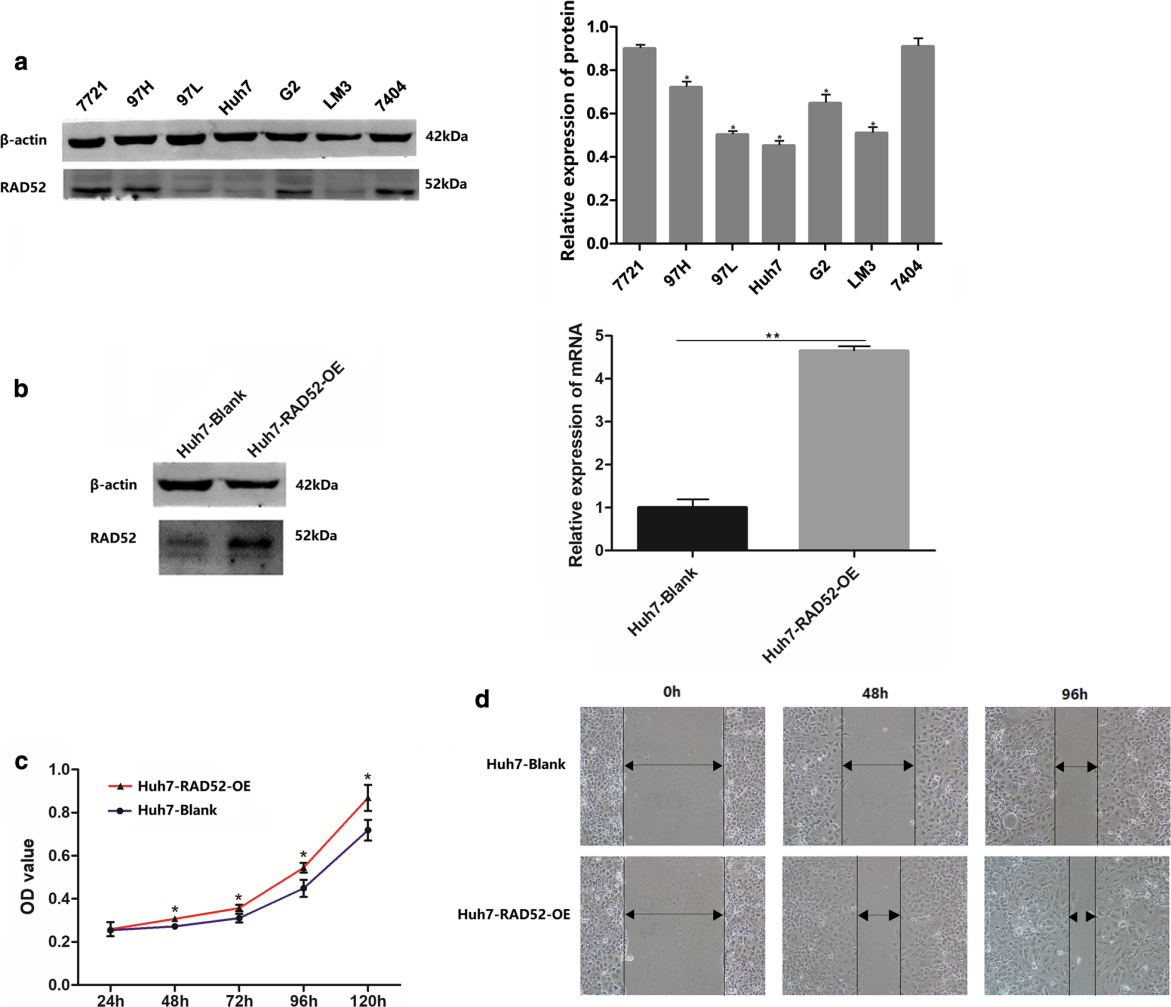
The functional role of RAD52 in HCC cell lines. **a** Expression level of RAD52 in human HCC cell lines including SMCC-7721 (7721), MHCC 97H (97H), MHCC 97L (97L), Huh7, HepG2 (G2), HCC-LM3 (LM3), and BEL7404 (7404) measured by Western blotting. Compared with the highest expression level of RAD52 in 7404, expression of RAD52 in Huh7 was lowest among these cells (P < 0.05, quantified by ImageJ). **b** Western blotting and qRT-PCR were used to detect RAD52 expression in RAD52-overexpression (OE)-transfected Huh7 cells. **c** CCK-8 test suggested that RAD52-OE in Huh7 cells could promote cell proliferation (P < 0.05). **d** Scratch wound assay showed that RAD52-OE in Huh7 cells could promote cell migration

According to the average expression level of RAD52 in 7721, 97H, 97L, Huh7, G2, LM3, and 7404 HCC cell lines (Fig. 3a), Huh7 cells, which expressed the lowest RAD52 among these cell lines, were selected to assess the role of RAD52 in HCC by a lentiviral system with puromycin selection. And it lead to a RAD52 over-expression in Huh7 cells, which was detected by Western blotting and qRT-PCR (Fig. 3b). Compared with Huh7 cells (Blank control), Cell growth assay and Scratch wound assay were then used to test the effect of RAD52 on HCC cell proliferation and migration. As shown in Fig. 3c, d, RAD52 over-expression significantly promoted the proliferation and migration of Huh7 cells in vitro.

### GO and KEGG pathway enrichment analyses of RAD52 and its interactome

In GO annotation and pathway enrichment analyses, RAD52, as well as RAD51, XRCC6, and CFL1, has been found in different biological pathways (Additional file 2). RAD52 and its interactors were enriched for double-strand break repair, chromosome organization, and macromolecular complex subunit organization. RAD52, as well as RAD51, was primarily linked to DNA recombinase assembly, double-strand break repair via synthesis-dependent strand annealing, and macromolecular complex subunit organization. RAD52 and XRCC6 were correlated with double-strand break repair and non-recombinational repair. RAD52 and CFL1 participated in macromolecular complex subunit organization. Cell component enrichment analysis indicated that RAD52 and its interactome were predominant at nucleoplasm. Generally, RAD52 and XRCC6 were colocated at the nucleoplasm, nuclear lumen, and protein complex. RAD52 and RAD51 were colocated at the nucleoplasm, while RAD52 and CFL1 were found in the nuclear lumen. Regarding molecular function, RAD52 and its interactors were enriched in structure-specific DNA binding. In KEGG pathway enrichment analyses, RAD52 and RAD51 were linked to homologous recombination.

### Construction and analysis of PPI networks

The PPI network for RAD52 is shown in Fig. 6b, with the corresponding module being shown in Fig. 6c. The most significantly enriched functional module containing RAD52 was linked to double-strand break repair, homologous recombination, structure-specific DNA binding, telomere organization, non-recombinational repair, DNA recombinase assembly, and double-strand break repair via synthesis-dependent strand annealing (Fig. 6a).

### Protein–protein docking

The results of docking between RAD52 and its interactome are presented in Fig. 6d–f. The analysis was based on the E-total energy of binding, which reflects the opportunity to bind. The E-total energy for RAD52–RAD51, RAD52–CFL1, and RAD52–XRCC6 binding were − 1120.5, − 996.6, and − 902.1 kcal/mol, respectively. Molecular thermodynamics indicated that lower E-total energy was correlated with an easier and firmer binding structure [29]. The optimal binding site of CFL1 at RAD52 was on the opposite side of the RAD52–RAD51 and RAD52–XRCC6 binding sites (Fig. 6d–f). Furthermore, the hot spots (some crucial amino acids) in the protein complex, which might play a vital role during the interaction, were also detected by KFC2 server (https://mitchell-lab.biochem.wisc.edu/KFC_Server/).

### Impact of RAD51, XRCC6 and CFL1 in patients with HCC

mRNA expression of RAD51, XRCC6 and CFL1 was analyzed using the data from TCGA database (363 HCC tissues and 50 normal tissues). Further validation was performed in Chinese patients with HCC (70 HCC tissues and paired adjacent tissues).

**Fig. 4:**
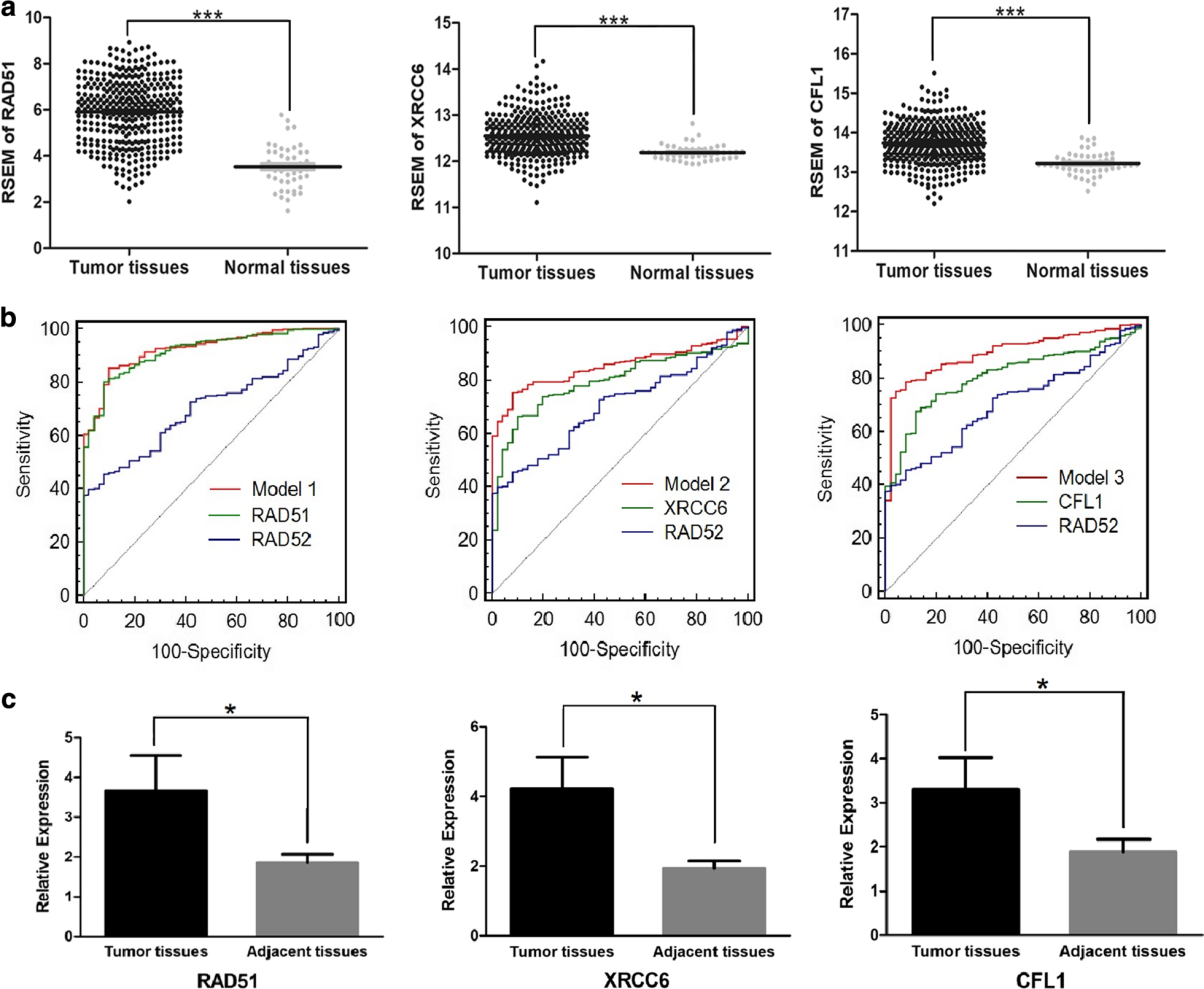
Data mining of RAD51, XRCC6 and CFL1. **a** RSEM of RAD51, XRCC6 and CFL1 in HCC tissues and normal tissues in the TCGA database. Higher expression of RAD51, XRCC6 and CFL1 was observed in HCC tissues ***P ≤ 0.001). **b** ROC curves of RAD52, RAD51, XRCC6, CFL1, and combined models in diagnosing HCC. Each AUC of the combined model was larger than that of RAD52. The AUC of the combined model (RAD52 and RAD51) was largest in all models (0.924). In each picture, the red solid ROC curve represented the combined model of RAD52 and another protein (the green solid). The blue solid ROC curve represents RAD52. The gray solid represents the reference line. **c** mRNA expression of RAD51, XRCC6, and CFL1 was confirmed in HCC tissues and adjacent tissues. Upregulated mRNA levels of RAD51, XRCC6, and CFL1 were observed in HCC tissues (*P ≤ 0.05)

**Fig. 5:**
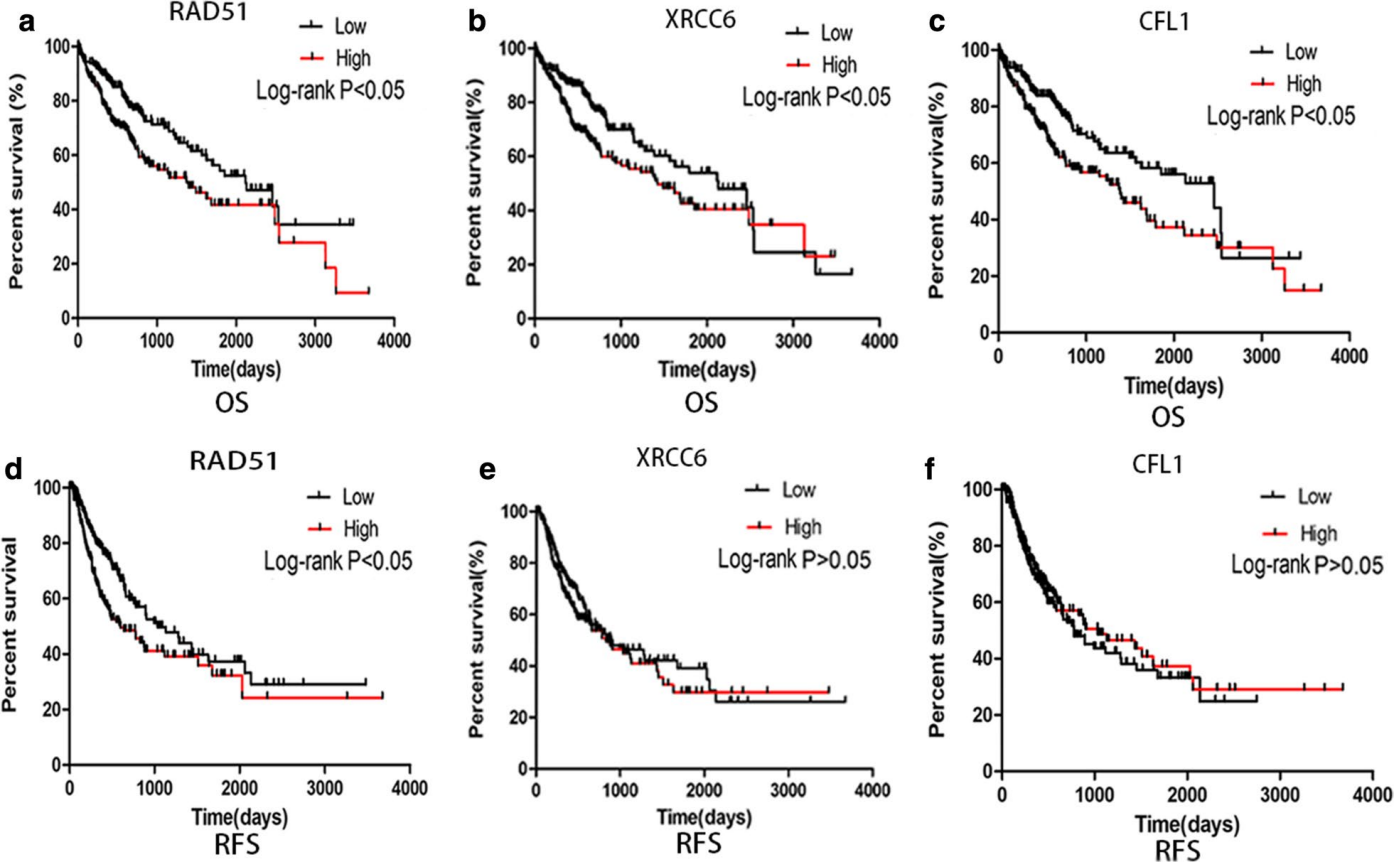
Survival curves of RAD51, XRCC6, and CFL1 in HCC. **a**–**c** Overall survival (OS) curves. Significant differences between high and low expression of RAD51, XRCC6, and CFL1 were found in OS. Higher RAD51, XRCC6, CFL1 have significantly poorer OS (*P ≤ 0.05). **d**–**f** Recurrence-free survival (RFS). Higher XRCC6 or CFL1 was not associated with shorter RFS (P > 0.05). However, higher RAD51 levels have significantly poorer RFS (*P ≤ 0.05)

The expression levels of RAD51, XRCC6 and CFL1 in HCC were higher than those in normal tissues or adjacent (peritumoral) tissues (Fig. 4a, c). Moreover, patients with high RAD51 expression were proven to have significantly poorer OS (*P ≤ 0.05) and shorter RFS (*P ≤ 0.05) than those with low RAD51 expression (Fig. 5a, d). The AUCs of RAD51, XRCC6, and CFL1 were 0.917, 0.795, and 0.808, respectively (Additional file 3).

**Fig. 6:**
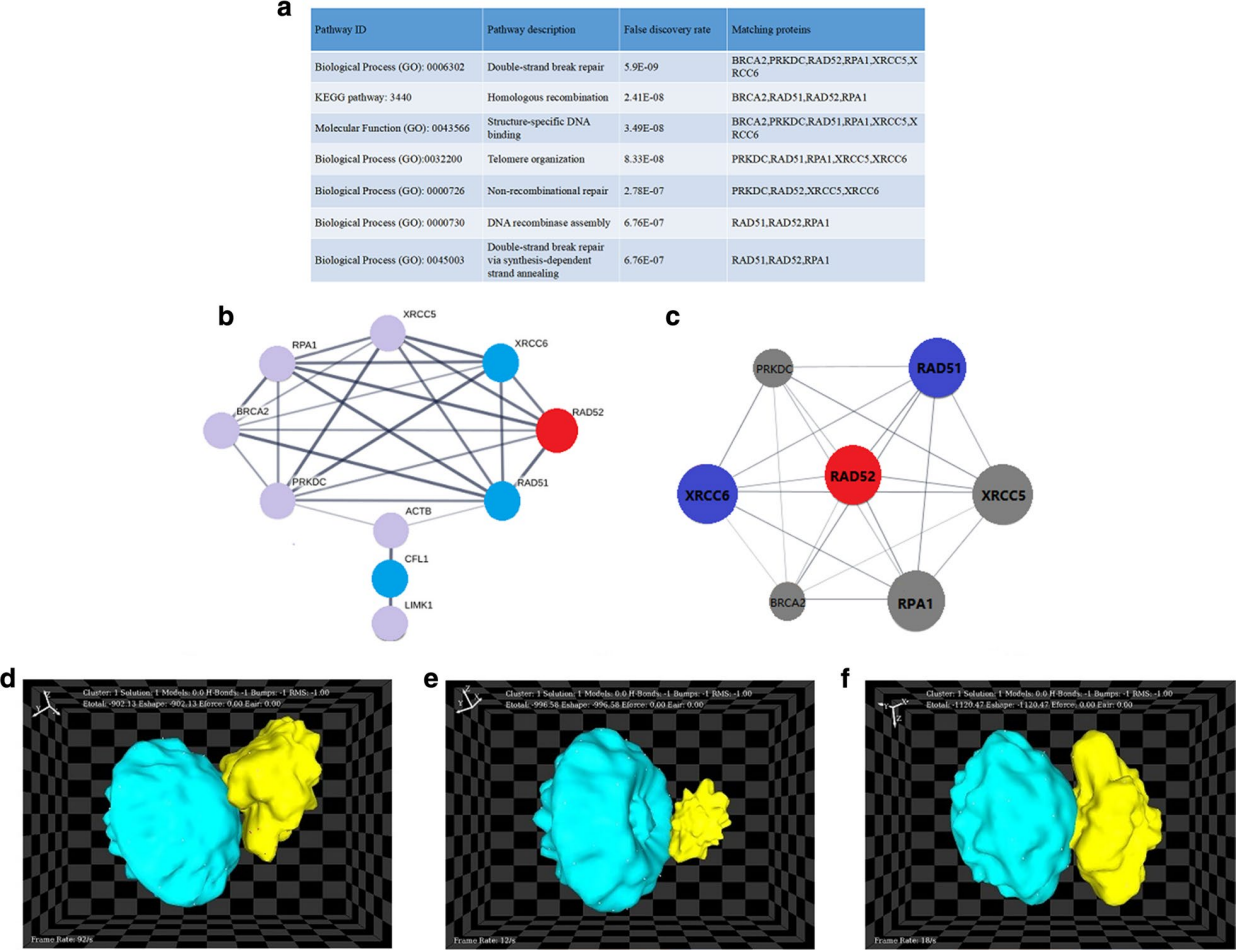
PPI network (with module analyses) and docking results between RAD52 and its interactors. The PDB numbers of RAD52, RAD51, XRCC6, and CFL1 were 1kn0, 5h1c, 1jey, 1q8g, respectively. (https://www.rcsb.org/) **a** The enrichment and pathways analysis. **b** PPI network of RAD52 and its interactome. The red node represents the core protein. Blue nodes represent the important DSB repair protein, while the gray nodes represent predicted functional node partners. **c** Core module for RAD52 and its interaction partners. **d** RAD52–XRCC6 complex, **e** RAD52–CFL1 complex, **f** RAD52–RAD51 complex

Three combined models were built to obtain better diagnosis efficiency for HCC (Fig. 6b). The ROC curve of model 1 represented the combined utility of RAD52 and RAD51 with an algorithm of Y = 0.775 * RAD52 + 1.531 * RAD51 − 9.826. The sensitivity and specificity were 85.4% and 90.0%, respectively. The AUC was 0.924, which was the highest among the three models. The ROC curve of model 2 represented the combined utility of RAD52 and XRCC6 with an algorithm of Y = 1.053 * RAD52 + 2.790 * XRCC6 − 39.070. The sensitivity and specificity were 78.5% and 86.0%, respectively, and the AUC was 0.853. The ROC curve of model 3 represented the combined utility of RAD52 and CFL1 with an algorithm of Y = 2.110 * RAD52 + 3.733 * CFL1 − 61.368. The AUC was 0.896, and the sensitivity and specificity were 78.8% and 92.0%, respectively.

Furthermore, the expression of RAD51 has been shown to be associated with the level of AFP in serum and clinical stage but not with other factors (Additional file 4). High expression of XRCC6 and CFL1 correlated with poor OS (*P ≤ 0.05) (Fig. 5b, c; Additional file 5).

## Discussion

There is little research on the function of RAD52 in HCC. The expression of RAD52 in HCC and its correlation with the clinicopathological features of HCC have not been thoroughly elucidated to date. We conducted an in-depth study of the relationship between RAD52 and HCC. RNA-seq data in the TCGA database were obtained to screen for HCC-specific mRNA expression alterations. RAD52 mRNA levels were increased in HCC. The results were validated by qRT-PCR analysis of HCC and its adjacent tissues. Moreover, the RAD52 protein level in HCC tissues was significantly higher than that in the adjacent tissues, which was confirmed by immunohistochemical analysis of paraffin sections and Western blotting with a large amount of clinical specimens. In addition, the expression of RAD52 protein was consistent with RAD52 mRNA. Since RAD52 plays an essential role in break-induced replication repair, this finding may explain the amplification of RAD52 in human cancers and why its inactivation disrupts cancer development [20, 31, 32].

Furthermore, we found that RAD52 mRNA expression showed a gender-specific association in the TCGA database. The RAD52 mRNA level was higher in female than in male patients. Epidemiological data suggested that women have a lower risk of HCC than men [33]. Some studies have suggested that this effect might be due to the stimulatory effects of androgen and the protective effects of estrogen [34]. However, we did not obtain the same result in the Chinese population. Limitation of the sample quantity and single race may cause this difference. Admittedly, a larger sample is necessary to confirm this result. Interestingly, the expression of RAD52 varied at different ages in Chinese patients. Patients’ age and postoperative complications are more relevant for the treatment (e.g., liver resection) than other risk factors, especially for patients over 70 years of age [35]. In contrast, the prognosis of patients below 70 years of age is significantly better, and a 5-year survival rate above 50% could be shown [36]. In Chinese populations, the expression of RAD52 was significantly higher in patients older than 45 years. This finding implied that an increase in age may induce an elevated expression of RAD52, which may enhance the tumor proliferation capacity and lead to a poor prognosis [36, 37]. In vitro, our HCC cell line studies suggested that over-expressed RAD52 significantly promoted the proliferation and migration of HCC cells.

To further explore the functional mechanism of RAD52 in HCC, we also investigated several genes that closely interact with RAD52 in DSB repair. RAD51 and RAD52 are two key proteins in homologous pairing and strand exchange during DSB-induced HR [37–39]. RAD51-mediated HR has critical roles in restarting stalled or collapsed replication forks [40]. Yeast Rad51 (ScRad51) and mammalian RAD51 play a central role in HR by forming nucleoprotein filaments on ssDNA that perform the homology search and strand exchange reactions [41–43]. ScRad52 is required for ScRad51 focus formation in yeast, and ScRad52 is one of the accessory factors to ensure the proper assembly of ScRad51 filaments [31, 44]. In mammals, RAD52 has retained similar biochemical activities as ScRAD52 in vitro, suggesting a similar function as ScRAD52 [4, 31]. It has been found that mammalian RAD51 and RAD52 function together in recombinational repair of DSBs in vivo and that RAD52 can enhance the strand exchange activity of RAD51 [45, 46]. Our results also showed an increase in both RAD51 and RAD52 protein in HCC. It has been reported that human RAD52 plays a role in HCC by forming a complex with its associated factors [47, 48]. In our study, there was no significant positive correlation between RAD51 and RAD52 expression, suggesting different roles of RAD52, depending on the levels of RAD51. Abnormal expression of RAD51 and RAD52 can contribute to genomic instability and tumor progression [49]. Our results demonstrated that overexpression of RAD51 and RAD52 could affect the development of HCC. Since the proper balance and regulation of DNA repair pathways would be predicted to be critical for the maintenance of genomic integrity, the interrelationship of different DNA repair pathways in HCC warrants further study.

Nonhomologous end-joining (NHEJ) is another important pathway of DSB repair [50]. X-ray repair cross complementing 6 (XRCC6/KU70) is the critical NHEJ factor [51]. Several studies proposed that XRCC6 inhibits HDR and SSA, two pathways of HR. In contrast, RAD52 enhanced HR [37, 38]. Some studies have shown that RAD52 competes with XRCC6 for binding to S-region DSB ends, and RAD52 facilitates Ku-independent DSB repair as well [52]. This result indicated that RAD52 competed with XRCC6 during DSB repair. Zhang et al. [53] demonstrated that XRCC6 expression was significantly increased in HCC, and its expression was significantly correlated with gender and maximal tumor size, as demonstrated by clinicopathological analysis. In our study, the expression of XRCC6 was significantly upregulated in HCC tissues. The roles of XRCC6 and RAD52 in HCC have not been thoroughly elucidated to date.

Cofilin (CFL1) is one of the proteins in the actin depolymerization factor/cofilin family that is responsible for cell motility [54]. Some studies suggest that CFL1 can influence the radiosensitivity of cells by altering DNA repair capacity and is involved in cancer cell migration, invasion, and metastasis [28, 38]. Our previous study suggested that CFL1 was upregulated in HCC, which may interact with H2AX mediating the DSB repair pathway [28]. Downregulation of RAD51 and XRCC6, two pivotal proteins that mediate NHEJ and HR, was detected in irradiated cofilin overexpressed cells [37, 51]. Downregulation of RAD51 and XRCC6 may suppress NHEJ and HR, even though normal Rad52-ssDNA complexes can be formed [38]. Interestingly, in our study, the increased expression of RAD51, RAD52, XRCC6 and CFL1 was obtained in HCC. The results indicated that these genes might cooperate in different ways in HCC pathogenesis.

The GO enrichment analysis also revealed that RAD52, RAD51, and XRCC6 were correlated with DSB repair. The KEGG pathway analysis also suggested that RAD52 and RAD51 were associated with homologous recombination. Due to the vital role that RAD52 is involved in different DSB repair pathways, RAD52 might be an important core protein mediating DSB repair. The high expression of RAD52 in HCC might suggest that it was abnormal in DSB repair in HCC. Interestingly, as for CFL1, the PPI network did not reveal a direct interaction between RAD52 and CFL1. Our network results suggested that they were connected by another protein, known as actin beta (ACBT).

To understand RAD52 and its interactions with other DNA repair proteins, as well as generating as many near-native complex structures (hits) as possible, protein docking calculations were performed. The RAD52–RAD51 complex has the lowest E-total energy among RAD52–RAD51, RAD52–CFL1, and RAD52–XRCC6 complex. This finding indicated firm binding between RAD52 and RAD51. Furthermore, the binding locations of these three proteins to RAD52 differed. The binding sites of RAD51 and XRCC6 to RAD52 were on the same side, which was opposed to the RAD52–CFL1 binding site. The different binding sites may be related to the different biological activities of RAD52.

Although the overexpression of RAD52 was most predictive of poor RFS in melanoma, high RAD52 was not correlated with poor OS and short RFS in HCC [55]. This finding suggested that RAD52 may have different effects on OS or RFS in different cancers, which needs to be further studied. For the ROC curve, the AUC of RAD52 was 0.704 (> 0.500), indicating that RAD52 is valuable and practical for the diagnosis of HCC. Furthermore, combining models of RAD52 with other genes (RAD51, XRCC6, CFL1) could enhance the diagnostic ability of RAD52 for HCC (*P ≤ 0.05). Larger AUCs with greater sensitivity and specificity were obtained in three combined models, especially model 1. A better assessment of model 1 than RAD52 may be associated with the close cooperation between RAD52 and RAD51 in DSB-induced HR. Models 2 and 3 may associate with the function of XRCC6 and CFL1 in NHEJ. Furthermore, higher RAD51, XRCC6 and CFL1 were correlated with poorer OS. Moreover, higher RAD51 was correlated with shorter RFS. These results suggested the different roles of those proteins in HCC pathogenesis. Compared with existing HCC diagnostic markers such as AFP (The sensitivity and specificity were 56.1% and 88.1%, while the AUC of AFP was 0.775) [56], RAD52 revealed a higher sensitivity (61.2%), while the specificity of RAD52 (71.0%) was lower that of AFP. Optimistically, ROC curves of combined models of RAD52 and its interactors had higher sensitivity and specificity (as well as AUC) than that of single AFP, except the comparable specificity of model 2 and AFP (86.0% vs 88.1%). The results of our study establish a foundation for the multigene prediction and diagnosis of HCC.

## Conclusions

RAD52 was upregulated in HCC tissues, and higher RAD52 was positively correlated with older age. The AUC of RAD52 was 0.704. Over-expressed RAD52 significantly promoted the proliferation and migration of HCC cells. Some key genes (RAD51, XRCC6 and CFL1) of DNA damage repair were increased in HCC. These genes might cooperate with RAD52 in HCC pathogenesis. The analysis of protein–protein interactions showed that the interactors have different binding sites and binding patterns according to different biological functions. An algorithm combining ROC between those proteins and RAD52 could provide greater specificity and sensitivity for HCC screening. Our findings suggest that RAD52 may play a vital role in HCC pathogenesis and may serve as a potential molecular target of HCC diagnosis and treatment. The multigene prediction and diagnosis of HCC are valuable. Further studies are warranted to obtain a better understanding of RAD52 function in HCC. The results of this study will be useful for HCC prevention and treatment.

## Supplementary information

**Additional file 1.** Molecular docking total energy values for RAD52 and its interactors with hot spot analyses.

**Additional file 2.** Gene ontology (GO) analyses and Kyoto Encyclopedia of Genes and Genomes (KEGG) pathway enrichment analyses of RAD52 and its node genes.

**Additional file 3.** Sensitivity and specificity to diagnosis HCC with different genes.

**Additional file 4.** Correlation between the factors and clinicopathologic characteristics in HCC in TCGA dataset (n = 363).

**Additional file 5.** The number of dead or alive patients, the number of patients with high or low RAD52, RAD51, XRCC6 and CFL1 expression, and values of log-ranktest and Gehan–Breslow–Wilcoxon from the Kaplan–Meier analysis in TCGA database.

## Data Availability

The datasets generated and/or analysed during the current study are available in TCGA LIHC dataset, https://xenabrowser.net/.

## Abbreviations

RAD52: radiation sensitive 52
RAD51: radiation sensitive 51
XRCC6: X-ray repair cross complementing 6
CFL1: cofilin
ACBT: actin beta
HCC: hepatocellular carcinoma
TCGA: The Cancer Genome Atlas
AUC: area under the receiver operating characteristic curve
OS: overall survival
RFS: recurrence-free survival
DSB: DNA double-strand break
HR: homologous recombination
NHEJ: nonhomologous end-joining
AFP: alpha-fetoprotein
RNA-seq: RNA sequencing
LIHC: liver hepatocellular carcinoma
RSEM: RNA-seq by expectation-maximization
PPI: protein–protein interaction
MCODE: molecular complex detection
PDB: Protein Data Bank
ROC: receiver operating characteristic curve
HBsAg: hepatitis B surface antigen
ALT: glutamic-pyruvic transaminase
AST: aspartate transaminase
OD: optical density
