# Supplementary material for: Evaluating the role of RAD52 and its interactors as novel potential molecular targets for hepatocellular carcinoma
Source: Cancer Cell Int. 2019 Nov 6;19:279. doi: 10.1186/s12935-019-0996-6 (PMC6836504; doi:10.1186/s12935-019-0996-6)
Supplement: Supplementary file 1 — Additional file 1. Molecular docking total energy values for RAD52 and its interactors with hot spot analyses. [file 12935_2019_996_MOESM1_ESM.docx]

| **Additional file 1.** Molecular docking total energy values for RAD52 and its interactors with hot spot analyses. | | | | |
| --- | --- | --- | --- | --- |
| **target proteins** | **the name of PDB files** | Etotal kcal·mol-1 | **Hot spots of target protein** | **Hot spots of RAD52** |
| RAD51 | 5h1c | -1130.96 | / | / |
| CFL1 | 1q8g | -996.58 | / | / |
| XRCC6 | 1jey | -573.03 | ARG(218),ASP(219),ILE(376),THR(380) | PRO(198),GLU(202),GLU(201),TYR(205),GLN(37) |
| RAD52: Radiation sensitive 52; RAD51: Radiation sensitive 51; XRCC6: X-ray repair cross complementing 6; CFL1: cofflin; PDB: Protein Data Bank. Predicted hot spots were amino acids. | | | | |
