# Supplementary material for: Evaluating the role of RAD52 and its interactors as novel potential molecular targets for hepatocellular carcinoma
Source: Cancer Cell Int. 2019 Nov 6;19:279. doi: 10.1186/s12935-019-0996-6 (PMC6836504; doi:10.1186/s12935-019-0996-6)
Supplement: Supplementary file 2 — Additional file 2. Gene ontology (GO) analyses and Kyoto Encyclopedia of Genes and Genomes (KEGG) pathway enrichment analyses of RAD52 and its node genes. [file 12935_2019_996_MOESM2_ESM.docx]

| **Additional file 2.** Gene ontology (GO) analyses and Kyoto Encyclopedia of Genes and Genomes (KEGG) pathway enrichment analyses of RAD52 and its node genes | | | | | |
| --- | --- | --- | --- | --- | --- |
| **Category** | **Pathway ID** | **Pathway Description** | **Gene count** | **Matching proteins** | **False discovery rate** |
| Biological process | GO.0006302 | double-strand break repair | 6 | BRCA2,PRKDC,RAD52,RPA1,XRCC5,XRCC6 | 0.000000174 |
| Biological process | GO.0032200 | telomere organization | 5 | PRKDC,RAD51,RPA1,XRCC5,XRCC6 | 0.000000991 |
| Biological process | GO.0000726 | non-recombinational repair | 4 | PRKDC,RAD52,XRCC5,XRCC6 | 0.00000167 |
| Biological process | GO.0000730 | DNA recombinase assembly | 3 | RAD51,RAD52,RPA1 | 0.00000232 |
| Biological process | GO.0045003 | double-strand break repair via synthesis-dependent strand annealing | 3 | RAD51,RAD52,RPA1 | 0.00000232 |
| Biological process | GO.0000723 | telomere maintenance | 4 | PRKDC,RPA1,XRCC5,XRCC6 | 0.0000499 |
| Biological process | GO.0000724 | double-strand break repair via homologous recombination | 4 | BRCA2,PRKDC,RAD52,RPA1 | 0.0000499 |
| Biological process | GO.0006303 | double-strand break repair via nonhomologous end joining | 3 | PRKDC,XRCC5,XRCC6 | 0.000132 |
| Biological process | GO.0051276 | chromosome organization | 6 | ACTB,BRCA2,PRKDC,RPA1,XRCC5,XRCC6 | 0.000875 |
| Biological process | GO.0006310 | DNA recombination | 4 | BRCA2,RPA1,XRCC5,XRCC6 | 0.00156 |
| Biological process | GO.0010332 | response to gamma radiation | 3 | BRCA2,PRKDC,RAD51 | 0.0017 |
| Biological process | GO.0032508 | DNA duplex unwinding | 3 | RAD51,XRCC5,XRCC6 | 0.0017 |
| Biological process | GO.0060249 | anatomical structure homeostasis | 4 | ACTB,PRKDC,XRCC5,XRCC6 | 0.00441 |
| Biological process | GO.0075713 | establishment of integrated proviral latency | 2 | XRCC5,XRCC6 | 0.00488 |
| Biological process | GO.0032481 | positive regulation of type I interferon production | 3 | PRKDC,XRCC5,XRCC6 | 0.00519 |
| Biological process | GO.0019042 | viral latency | 2 | XRCC5,XRCC6 | 0.00814 |
| Biological process | GO.0045087 | innate immune response | 5 | ACTB,CFL1,PRKDC,XRCC5,XRCC6 | 0.0175 |
| Biological process | GO.0045005 | DNA-dependent DNA replication maintenance of fidelity | 2 | BRCA2,RAD51 | 0.021 |
| Biological process | GO.0030097 | hemopoiesis | 4 | BRCA2,PRKDC,RPA1,XRCC5 | 0.0305 |
| Biological process | GO.0010165 | response to X-ray | 2 | BRCA2,RAD51 | 0.0383 |
| Biological process | GO.0006312 | mitotic recombination | 2 | RAD51,RPA1 | 0.0442 |
| Biological process | GO.0002520 | immune system development | 4 | BRCA2,PRKDC,RPA1,XRCC5 | 0.0445 |
| Biological process | GO.0043933 | macromolecular complex subunit organization | 6 | ACTB,BRCA2,CFL1,RAD51,RAD52,RPA1 | 0.045 |
| Cell component | GO.0070419 | nonhomologous end joining complex | 3 | PRKDC,XRCC5,XRCC6 | 0.00000481 |
| Cell component | GO.0043564 | Ku70:Ku80 complex | 2 | XRCC5,XRCC6 | 0.000149 |
| Cell component | GO.0005654 | nucleoplasm | 8 | ACTB,BRCA2,PRKDC,RAD51,RAD52,RPA1,XRCC5,XRCC6 | 0.00122 |
| Cell component | GO.0000783 | nuclear telomere cap complex | 2 | XRCC5,XRCC6 | 0.00308 |
| Cell component | GO.0000800 | lateral element | 2 | RAD51,RPA1 | 0.00308 |
| Cell component | GO.0044454 | nuclear chromosome part | 4 | ACTB,RAD51,XRCC5,XRCC6 | 0.00308 |
| Cell component | GO.0032993 | protein-DNA complex | 3 | RPA1,XRCC5,XRCC6 | 0.00606 |
| Cell component | GO.0000784 | nuclear chromosome, telomeric region | 2 | XRCC5,XRCC6 | 0.00966 |
| Cell component | GO.0000795 | synaptonemal complex | 2 | RAD51,RPA1 | 0.0129 |
| Cell component | GO.0031981 | nuclear lumen | 7 | ACTB,BRCA2,CFL1,PRKDC,RAD52,XRCC5,XRCC6 | 0.0203 |
| Cell component | GO.0043234 | protein complex | 7 | ACTB,BRCA2,PRKDC,RAD52,RPA1,XRCC5,XRCC6 | 0.0407 |
| Cell component | GO.0030863 | cortical cytoskeleton | 2 | ACTB,CFL1 | 0.0423 |
| Molecular function | GO.0043566 | structure-specific DNA binding | 6 | BRCA2,PRKDC,RAD51,RPA1,XRCC5,XRCC6 | 0.00000102 |
| Molecular function | GO.0003684 | damaged DNA binding | 4 | RAD51,RPA1,XRCC5,XRCC6 | 0.0000248 |
| Molecular function | GO.0003690 | double-stranded DNA binding | 4 | PRKDC,RAD51,XRCC5,XRCC6 | 0.000158 |
| Molecular function | GO.0008094 | DNA-dependent ATPase activity | 3 | RAD51,XRCC5,XRCC6 | 0.00168 |
| Molecular function | GO.0003697 | single-stranded DNA binding | 3 | BRCA2,RAD51,RPA1 | 0.00389 |
| Molecular function | GO.0042162 | telomeric DNA binding | 2 | XRCC5,XRCC6 | 0.0154 |
| Molecular function | GO.0008022 | protein C-terminus binding | 3 | RAD51,XRCC5,XRCC6 | 0.0163 |
| Molecular function | GO.0004003 | ATP-dependent DNA helicase activity | 2 | XRCC5,XRCC6 | 0.0209 |
| KEGG | 3440 | Homologous recombination | 4 | BRCA2,RAD51,RAD52,RPA1 | 0.000000144 |
| KEGG | 3450 | Non-homologous end-joining | 3 | PRKDC,XRCC5,XRCC6 | 0.00000344 |
| KEGG | 3460 | Fanconi anemia pathway | 3 | BRCA2,RAD51,RPA1 | 0.000166 |
| KEGG | 4810 | Regulation of actin cytoskeleton | 3 | ACTB,CFL1,LIMK1 | 0.00846 |
| KEGG | 5212 | Pancreatic cancer | 2 | BRCA2,RAD51 | 0.0222 |
| KEGG | 4666 | Fc gamma R-mediated phagocytosis | 2 | CFL1,LIMK1 | 0.0376 |
