# Supplementary material for: Evaluating the role of RAD52 and its interactors as novel potential molecular targets for hepatocellular carcinoma
Source: Cancer Cell Int. 2019 Nov 6;19:279. doi: 10.1186/s12935-019-0996-6 (PMC6836504; doi:10.1186/s12935-019-0996-6)
Supplement: Supplementary file 3 — Additional file 3. Sensitivity and specificity to diagnosis HCC with different genes. [file 12935_2019_996_MOESM3_ESM.doc]

| **Additional file 3.** Sensitivity and specificity to diagnosis HCC with different genes. | | | |
| --- | --- | --- | --- |
|  | AUC | Sensitivity (%) | Specificity (%) |
| RAD52 | 0.704 | 61.2 | 71.0 |
| RAD51 | 0.917 | 56.6 | 56.6 |
| XRCC6 | 0.795 | 73.8 | 80.0 |
| CFL1 | 0.808 | 74.1 | 80.0 |
| Model 1 | 0.924 | 85.4 | 90.0 |
| Model 2 | 0.853 | 78.5 | 86.0 |
| Model 3 | 0.896 | 78.8 | 92.0 |
| AUC: area under ROC curve; RAD52: Radiation sensitive 52; RAD51: Radiation sensitive 51; XRCC6: X-ray repair cross complementing 6; CFL1: cofflin; Three combined models were acquired using the following algorithms. Model 1: Y=0.775*RAD52+1.531*RAD51-9.826; Model 2: Y=1.053*RAD52+2.790*XRCC6-39.070; Model 3: Y=2.110*RAD52+3.733*CFL1-61.368. | | | |
