# Supplementary material for: Evaluating the role of RAD52 and its interactors as novel potential molecular targets for hepatocellular carcinoma
Source: Cancer Cell Int. 2019 Nov 6;19:279. doi: 10.1186/s12935-019-0996-6 (PMC6836504; doi:10.1186/s12935-019-0996-6)
Supplement: Supplementary file 4 — Additional file 4. Correlation between the factors and clinicopathologic characteristics in HCC in TCGA dataset (n = 363). [file 12935_2019_996_MOESM4_ESM.doc]

| **Additional file 4.** Correlation between the factors and clinicopathologic characteristics in HCC in TCGA dataset (n=363). | | | |
| --- | --- | --- | --- |
| Clinical features | Case | RAD51 level | p value |
|  |  | (RSEM; mean±SD) |  |
| **Sample** |  |  |  |
| LIHC | 363 | 5.9162±1.4047 | p<0.05 |
| Normal | 50 | 3.5313±0.9018 |  |
| **Age at diagnosis(years)** |  |  |  |
| ＞45 | 314 | 5.8771±1.3977 | p>0.05 |
| ≤45 | 48 | 6.1513±1.4498 |  |
| Unknown | 1 |  |  |
| **Gender** |  |  |  |
| Male | 246 | 5.9111±1.4601 | p>0.05 |
| Female | 117 | 5.9268±1.2867 |  |
| **The AFP in serum** |  |  |  |
| >20ng/ml | 129 | 6.3471±1.2344 | p<0.05* |
| ≤20ng/ml | 143 | 5.4300±1.3743 |  |
| Unknown | 91 |  |  |
| **Clinical stage** |  |  |  |
| I–II | 251 | 5.8250±1.3232 | p<0.05* |
| III–IV | 88 | 6.2812±1.5355 |  |
| Unknown | 24 |  |  |
| **Child-Pugh classification** |  |  |  |
| A | 213 | 5.8002±1.3478 | p>0.05 |
| B | 21 | 5.6140±1.3094 |  |
| C | 1 |  |  |
| Unknown | 128 |  |  |
| **Lymph node** |  |  |  |
| N0 | 246 | 5.9899±1.4033 | p>0.05 |
| N1 | 3 | 6.5873±0.8147 |  |
| Unknown | 114 |  |  |
| **Metastasis** |  |  |  |
| M0 | 260 | 6.0072±1.424 | p>0.05 |
| M1 | 4 | 5.3115±0.6785 |  |
| Unknown | 99 |  |  |
| TCGA: The Cancer Genome Atlas; LIHC: Liver Hepatocellular Carcinoma; HCC: hepatocellular carcinoma; RSEM: RNA-Seq by Expectation-Maximization; RAD51: RAD51 recombinase; AFP: Alpha Fetoprotein; SD: standard deviation. * p < 0.05. | | | |
