# Supplementary material for: Evaluating the role of RAD52 and its interactors as novel potential molecular targets for hepatocellular carcinoma
Source: Cancer Cell Int. 2019 Nov 6;19:279. doi: 10.1186/s12935-019-0996-6 (PMC6836504; doi:10.1186/s12935-019-0996-6)
Supplement: Supplementary file 5 — Additional file 5. The number of dead or alive patients, the number of patients with high or low RAD52, RAD51, XRCC6 and CFL1 expression, and values of log-ranktest and Gehan–Breslow–Wilcoxon from the Kaplan–Meier analysis in TCGA database. [file 12935_2019_996_MOESM5_ESM.doc]

| **Additional file 5.** The number of dead or alive patients, the number of patients with high or low RAD52, RAD51, XRCC6 and CFL1 expression, and values of Log-ranktest and Gehan-Breslow-Wilcoxon from the Kaplan-Meier analysis in TCGA database. | | | | | | | | | |
| --- | --- | --- | --- | --- | --- | --- | --- | --- | --- |
| **Protein** | **Prognosis** | **Expression** | | **Log-rank test** | | **Gehan-Breslow-Wilcoxon Test** | | **HR** | **95% CI of HR** |
|  |  | **Low** | **High** | **Chi square** | **p value** | **Chi square** | **p value** |  |  |
| RAD52 | Overall survival | 5.9342±0.5174  (n=179) | 6.9881±0.3101  (n=178) | 0.03218 | 0.8576 | 0.2462 | 0.6197 | 1.032 | 0.7304-1.459 |
|  | Recurrence-free  survival | 5.9535±0.4825  (n=157) | 6.9912±0.3197  (n=155) | 1.353 | 0.2448 | 0.1979 | 0.6565 | 1.220 | 0.8724-1.707 |
| RAD51 | Overall survival | 4.7635±0.8857  (n=181) | 7.0717±0.6934  (n=176) | 7.220 | 0.0072 | 8.362 | 0.0038 | 0.6207 | 0.4383-0.8789 |
|  | Recurrence-free  survival | 4.7478±0.8899  (n=161) | 7.0781±0.6859  (n=151) | 5.172 | 0.0230 | 8.678 | 0.0032 | 0.6747 | 0.4807-0.9471 |
| XRCC6 | Overall survival | 12.2149±0.2348  (n=179) | 12.8882±0.3228  (n=178) | 4.472 | 0.0345 | 7.072 | 0.0078 | 0.6885 | 0.4871-0.9731 |
|  | Recurrence-free  survival | 12.2170±0.2357  (n=159) | 12.8828±0.3290  (n=153) | 0.7400 | 0.3897 | 1.221 | 0.2691 | 0.8628 | 0.6164-1.208 |
| CFL1 | Overall survival | 13.3239±0.3179  (n=181) | 14.1553±0.3229  (n=176) | 7.903 | 0.0049 | 8.507 | 0.0035 | 0.6073 | 0.4289-0.8598 |
|  | Recurrence-free  survival | 13.3204±0.3203  (n=166) | 14.1499±0.3286  (n=146) | 0.04926 | 0.8244 | 0.01514 | 0.9021 | 1.039 | 0.7409-1.457 |
| RAD52: Radiation sensitive 52; RAD51: Radiation sensitive 51; XRCC6: X-ray repair cross complementing 6; CFL1: cofflin; TCGA: The Cancer Genome Atlas; HR: Hazard ratio. | | | | | | | | | |
